# Physiology of the Brain

**Published:** 1874-10

**Authors:** H. R. Bigelow


Physiology of the Brain.—*By H. R. Bigelow, M.D.*—To sum up, then, in brief, some of the facts which, as skillful diagnosticians, we must be familiar with at the bedside, it is to be borne in mind—

1st. That grave cerebral lesions may exist without characteristic semeiology.

2d. That slight lesions may occasion a train of symptoms indicative of serious organic disease.

3d. That a paralysis is caused by the irritation transmitted by the cerebral lesion, inhibiting certain functional actions, and not by the mere circumscribed pressure.

4th. That a lesion in the middle line—i. e., affecting both sides of the optic thalami, striate bodies, etc.—may produce paralysis of but one side of the body.

5th. That a paralysis may exist on the same side with the lesion.

6th. That unilateral convulsions are much oftener associated with lesions of the right brain than with the left.

7th. That hysterical paralysis, etc., depends oftenest upon disorganized action of the right brain.

8th. That aphasia, agraphia, a mechanical impairment of speech and mental alienation, are usually associated with disease of the left brain.

9th. That the right brain is more capable than the left of producing paralysis on the same or on the opposite side of the body.

10th. Optic neuritis as a cause of amaurosis depends more often on a disease of the right than on a disease of the left.

11th. The symptoms may indicate a double lesion when one is present and conversely.—*Buffalo Medical and Surgical Journal.*

